# Transcriptome-wide identification and screening of WRKY factors involved in the regulation of taxol biosynthesis in *Taxus chinensis*

**DOI:** 10.1038/s41598-018-23558-1

**Published:** 2018-03-26

**Authors:** Meng Zhang, Ying Chen, Lin Nie, Xiaofei Jin, Weifang Liao, Shengying Zhao, Chunhua Fu, Longjiang Yu

**Affiliations:** 1 Institute of Resource Biology and Biotechnology, Department of Biotechnology, College of Life Science and Technology, Huazhong University of Science and Technology, No.1037 Luoyu Road, Wuhan, 430074 Hubei P. R. China; 2 Key Laboratory of Molecular Biophysics Ministry of Education, College of Life Science and Technology, Huazhong University of Science and Technology, No.1037 Luoyu Road, Wuhan, 430074 P. R. China

## Abstract

WRKY, a plant-specific transcription factor family, plays important roles in pathogen defense, abiotic cues, phytohormone signaling, and regulation of plant secondary metabolism. However, little is known about the roles, functions, and mechanisms of WRKY in taxane biosynthesis in *Taxus* spp. In this study, 61 transcripts were identified from *Taxus chinensis* transcriptome datasets by using hidden Markov model search. All of these transcripts encoded proteins containing WRKY domains, which were designated as TcWRKY1–61. After phylogenetic analysis of the WRKY domains of TcWRKYs and AtWRKYs, 16, 8, 10, 14, 5, 7, and 1 TcWRKYs were cladded into Group I, IIa–IIe, and III, respectively. Then, six representative TcWRKYs were selected to classify their effects on taxol biosynthesis. After MeJA (methyl jasmonate acid) and SA (salicylic acid) treatments, all of the six *TcWRKYs* were upregulated by MeJA treatment. *TcWRKY44* (IId) and *TcWRKY47* (IIa) were upregulated, whereas *TcWRKY8* (IIc), *TcWRKY20* (III), *TcWRKY26* (I), *TcWRKY41* (IIe), and *TcWRKY52* (IIb) were downregulated by SA treatment. Overexpression experiments showed that the six selected TcWRKYs exerted different effects on taxol biosynthesis. In specific, TcWRKY8 and TcWRKY47 significantly improved the expression levels of taxol-biosynthesis-related genes. Transcriptome-wide identification of WRKY factors in *Taxus* not only enhances our understanding of plant WRKY factors but also identifies candidate regulators of taxol biosynthesis.

## Introduction

Taxol is the most effective chemotherapy medication used to treat many cancers, including ovarian, breast, lung, Kaposi sarcoma, cervical, and pancreatic^1^. Taxol biosynthesis is complicated and involves approximately 19–20 steps of enzyme reaction catalyzed from geranylgeranyl pyrophosphate^2–4^. Most of the biosynthesis genes were isolated and their functions investigated in the past decades. However, only a few studies focused on the regulation mechanisms underlying the biosynthesis of these secondary metabolites. Recently, 5′ flanking sequences of several enzyme genes, including 10-deacetylbaccatin III-10-β-O-acetyltransferase (*DBAT*), taxadiene synthase (*TS*), phenylpropanoyl transferase (*BAPT*), taxoid 5α-hydroxylase (*T5H*), taxoid 7α-hydroxylase (*T7H*), and taxoid 10α-hydroxylase (*T10H*), have been submitted to public databases. Notably, W-box, the specific *cis*-element binding with WRKY proteins, was detected in all of these promoter regions, indicating that WRKY plays important roles in the regulation of taxol biosynthesis^5,6^.

WRKY, which is named after the conserved WRKYGQK motif in the WRKY domain, constitutes a dominant family of transcription factors in plants. All of the known WRKY factors could be divided into Group I to III according to the numbers of WRKY domains and their types of zinc finger motifs^7^. Group I has two WRKY domains, whereas Group II and III contain only one WRKY domain. Furthermore, Group II and III are distinguished because of their different zinc finger motifs, i.e., C_2_H_2_ (C-X_4–5_-C-X_22–23_-H-X_1_-H) type in Group II WRKYs, whereas C_2_HC (C-X_7_-C-X_23_-H-X_1_-C) type in Group III. Commonly, Group II WRKY factors could be further divided into five subgroups (i.e., Groups IIa–IIe) based on the specific characteristics of the WRKY domains^8–10^. Recently, five novel WRKY proteins have been observed to contain three or four WRKY domains from 43 plants by genome-wide identification, which is rare in plants^11^.

Currently, WRKY transcription factors have become the most pivotal transcription factors in plants because of their indispensable roles in regulating various physiological processes, such as biotic and abiotic stress^12^, signal molecule delivery^13^, and plant senescence and biosynthesis of secondary metabolites^14,15^. For example, GaWRKY1 regulates the sesquiterpene synthase *CAD1* ((+)-*δ-cadinene synthase-A*) gene, which is involved in gossypol pathway regulation in *Gossypium arboretum*^16^. AaWRKY1 regulates *amorpha-4,11-diene synthase*, which is involved in artemisinin biosynthesis in *Artemisia annua*^17^. We also observed that TcWRKY1 could specifically interact with the promoter of the *DBAT* gene^6^. All of these results indicate that WRKY factors play important roles in taxol biosynthesis.

Nowadays, high-throughput screening of regulation factors from various omics datasets has become economical and effective for researchers. In wheat (*Triticum aestivum* L.), 48 putative drought-induced WRKY genes were initially identified from a transcriptome, and TaWRKY33 was found to serve excellent functions in enhancing the drought tolerance of wheat^18^. In *Arabidopsis thaliana*, analysis of RNA sequencing data revealed that AtWRKY46, AtWRKY54, and AtWRKY70 play global roles in promoting brassinosteroid-mediated gene expression and inhibiting drought-responsive genes^19^. In summary, omics analysis has become an effective method to screen important transcription factors.

In this study, 61 TcWRKYs were identified by transcriptome-wide identification from *Taxus chinensis*. Multiple sequence alignment, motif analysis, and phylogenetic analysis were conducted to analyze the evolutionary relationship of WRKY factors between *Taxus* and angiosperms. Then, six TcWRKYs were selected for functional studies to identify their relationships with taxol biosynthesis. Our work enhances the understanding of WRKY factors in gymnosperm and identifies several effective candidate regulators of taxol biosynthesis.

## Materials and Methods

### Transcriptome-wide identification of *TcWRKYs* in *Taxus chinensis*

Previously, three pairs of samples, MeJA- (Methyl Jasmonate acid), GA-treated and NA/CA cells (Accession Numbers: SRR1343578, SRR1339474, and GSE28539), were high-throughout sequenced^20,21^. In MeJA-treated and NA cells, taxol and taxanes contents were significantly higher than control cells, while there was no difference between GA-treated and control cells. Thus, these datasets would help us analyze the relationships of expression patterns of WRKY factors with taxol biosynthesis. To improve the efficiency of gene screening, all reads of these transcriptomes were re-assembled by Trinity, totally 34 Gbp (2 Gbp for MJ-, 16 Gbp for GA- and NA/CA cells respectively)^22,23^. Finally, 67,147 unigenes were obtained with N50 value of 1,552 bp. Then, the HMM model of WRKY (PF03106) were retrieved from the Pfam database (http://pfam.sanger.ac.uk). After redundant sequences were removed, the HMMER program was used to identify the WRKYs, with an e-value cutoff of 1e-5. These unigenes containing two WRKY domains were separated as Group I, the others with only WRKY domain need further analysis to divide. Moreover, the WRKYs of *Arabidopsis thaliana* were downloaded from PlantTFDB database (http://planttfdb.cbi.pku.edu.cn/).

### Classification and phylogenetic analysis of conserved sequence of TcWRKY genes

The AtWRKYs protein sequences were downloaded from TAIR (http://www.arabidopsis.org/), and pfam database was downloaded at http://pfam.xfam.org/. Hmmscan programe of HMMER package was used to identify the conserved domains of AtWRKYs and TcWRKYs with E cut-off 1e-5. MEME was used to generate the motif logo of AtWRKYs and TcWRKYs. Motif LXXLL (or LXLXLX) and HARF (RTGHARFRR (A/G) P) were found manually.

The conserved sequences of *A*. *thiatina* were selected to build the phylogenetic tree. Multiple alignment was conducted by ClustalW with identity protein weight matrix. Phylogenetic analysis was performed with a neighbor-joining (NJ) method by using bootstrapping with 1000 repeats and Possion Correction model with 1000 resamplings in MEGA 5.0. Phylogenetic tree was modified by FigTree V1.4.2.

### Plant hormones treatment

*In vitro* long-term subcultured cells of *Taxus chinensis* were maintained on 62# medium containing 0.5 mg/L 6-BA, 0.5 mg/L 6BA, and 0.5 mg/L 2,4-D under two-day conditions. Then, 6 g cells were suspended in 50 mL fresh 62# medium, shaked at 25 °C with 125 rpm for 48 h dark period. Then, the final concentration of 0.1 mmol/L MeJA and 2.5 mmol/L SA were added into the liquid medium. These samples were harvested in liquid N_2_ after treated at 0, 1, 3 and 6 h for gene expression analysis.

### Gene cloning and construction of TcWRKY Overexpression Vectors

The total RNA of *Taxus chinensis* cells was reverse-transcribed to cDNA by reverse transcription kit (Thermo Scientific, USA). Specific primers were designed based on our transcriptome data (Supplementary Table S1). PCR procedures were as following: 96 °C for 5 min; 94 °C for 40 s, 52 °C for 40 s, 72 °C for 30 s, 30 cycles; 72 °C for 10 min, 16 °C for 10 min. The PCR products were subcloned into pMD18-T (TaKaRa, Japan) for sequencing.

*TcWRKY*-T plasmids were isolated by Plasmid Mini Kit (TIANGEN, China). TcWRKY8, TcWRKY20, TcWRKY26, TcWRKY41, TcWRKY44 and TcWRKY47 were digested with *Sma* I and *Bam*H I while TcWRKY52 were digested with *Bam*H I and *Sac* I. Then TcWRKY8, TcWRKY26, TcWRKY41, TcWRKY47 and TcWRKY52 were cloned into pBI121 while TcWRKY20 and TcWRKY44 were cloned into pCAMBIA1303 vectors. They were all placed under the control of the CaMV 35S promoter.

### Quantificational real-time polymerase chain reaction

The overexpression of TcWRKYs was analyzed by qRT-PCR with SYBR Green II method. The reaction system contained 5 μl SYBR Premix buffer, 0.5 μl each of the primers and 1 μl template and 3 μl ddH_2_O. The thermal profile for qRT-PCR was as follows: holding stage: 95 °C for 5 min; cycling stage: 95 °C for 10 s, 52 °C for 10 s, 72 °C for 15 s, 40 cycles; melting stage: 95 °C for 1 min, 65 °C to 95 °C 0.3 °C increase per cycle for 15 s. Each reaction was run in triplicate to obtain the average value and 2^−ΔΔCt^ method was applied for the analysis gene expression.

### The transformation assay in Transgenic *Taxus chinensis* cells

6 g *Taxus chinensis* CA cells were suspended in 50 mL fresh 62# medium. Then the *Agrobacterium tumefaciens* strain LBA4404 containing the TcWRKY-overexpressing constructs were added to ensure the value of absorbance were 0.6 in these mediums. Finally, the concentration of 1 mol/L AS (Acetosyringone, Biofroxx, German) was added into the liquid medium. They were shaked at 25 °C with 125 rpm for 48 h dark period. LBA4404 with the pBI121 or pCAMBIA1303-overexpressing constructs were as the control group.

## Results

### Identification of TcWRKYs from *T. chinensis* transcriptome datasets

For the identification of WRKY genes from *T*. *chinensis*, all known WRKY of *A*. *thaliana* and *Oryza sativa* were used as queries to perform local BLASTP search on the *T*. *chinensis* transcriptome datasets. Then, the obtained sequences were submitted to the NCBI-CDD web server (http://www.ncbi.nlm.nih.gov/cdd/) to analyze their conserved protein domain. Finally, a total of 61 unique TcWRKY genes encoding conserved WRKY domains were identified.

### Phylogenetic analysis of WRKY domains

Phylogenetic analysis was performed using all putative 61 TcWRKY proteins in *T*. *chinensis* and 71 AtWRKY proteins in *A*. *thaliana* to categorize and investigate the evolutionary relationships of TcWRKY genes.

**Figure 1:**
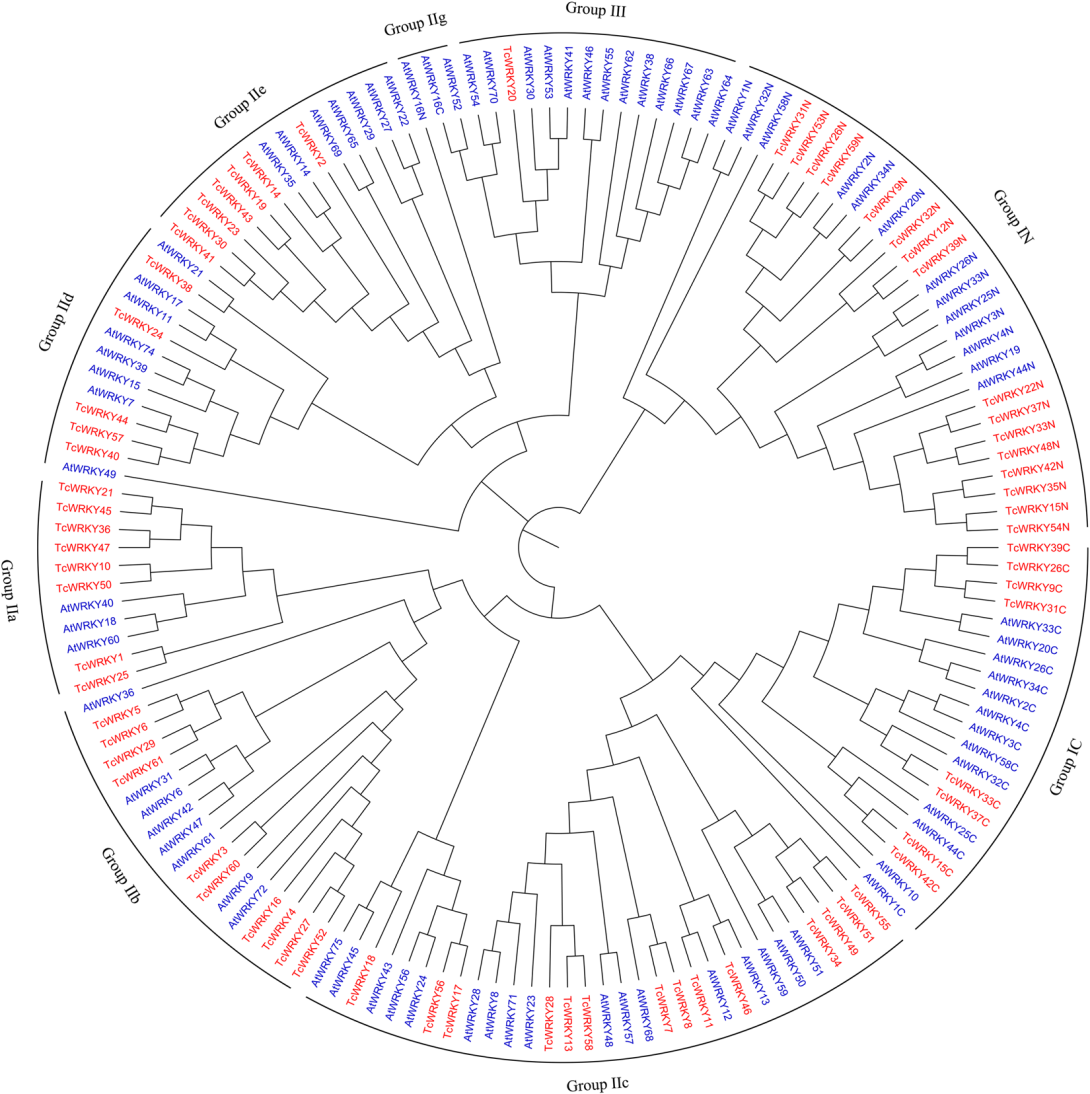
Phylogenetic tree of WRKY domains of TcWRKYs and AtWRKYs. TcWRKYs were red and AtWRKYs were blue. The names with N or C represented for N- and C-terminal WRKY domain of Group I respectively. All TcWRKYs were included in Supplementary file 2, and AtWRKYs were obtained from TAIR (http://www.arabidopsis.org/).

Results of phylogenetic analysis (Fig. 1) showed that all selected WRKYs, including TcWRKY proteins, could be categorized into three groups, i.e., Group I, II, and III. Among these TcWRKYs, 16 were categorized as Group I. Meanwhile, only 5 of 16 were full length after online BLAST. However, eight TcWRKYs encoded two WRKY domains (called Group IN and IC) and C_2_H_2_ type of zinc finger motif. Then, 44 TcWRKYs were assigned to Group II, which contains a single WRKY domain and C_2_H_2_ type of zinc finger motif. Furthermore, these 44 Group II TcWRKYs were classified into five subgroups, i.e., 8 in Group IIa, 10 in Group IIb, 14 in Group IIc, 5 in Group IId, and 7 in Group IIe. Notably, these five subgroups could be summed up to three branches, i.e., IIa + b, IIc, and IId + e; the same results were reported in many other plants^13^. Finally, only TcWRKY20 was categorized as Group III, which had one WRKY domain and C_2_HC type of zinc finger motif. These results are in accordance with the results of *Arabidopsis* and other plants, indicating that WRKY differentiated completely before evolutionary bifurcation of gymnosperm and angiosperm^8,24,25^.

AtWRKY19 (Group I), AtWRKY16 (Group IIg), and AtWRKY52 (Group IIg) appear to be unique. These results are similar to previous reports because only three AtWRKYs were R protein-WRKYs (both resistance (R) proteins and WRKY transcription factors) in *Arabidopsis*^26,27^. Moreover, AtWRKY16 and AtWRKY52 could be separated as Group IIg WRKYs, which is rare in plants, although AtWRKY16 contains two WRKY domains. According to our phylogenetic analysis, no Group IIg WRKY was detected in *T*. *chinensis*.

### Sequence alignment of WRKY domains of TcWRKYs

**Figure 2:**
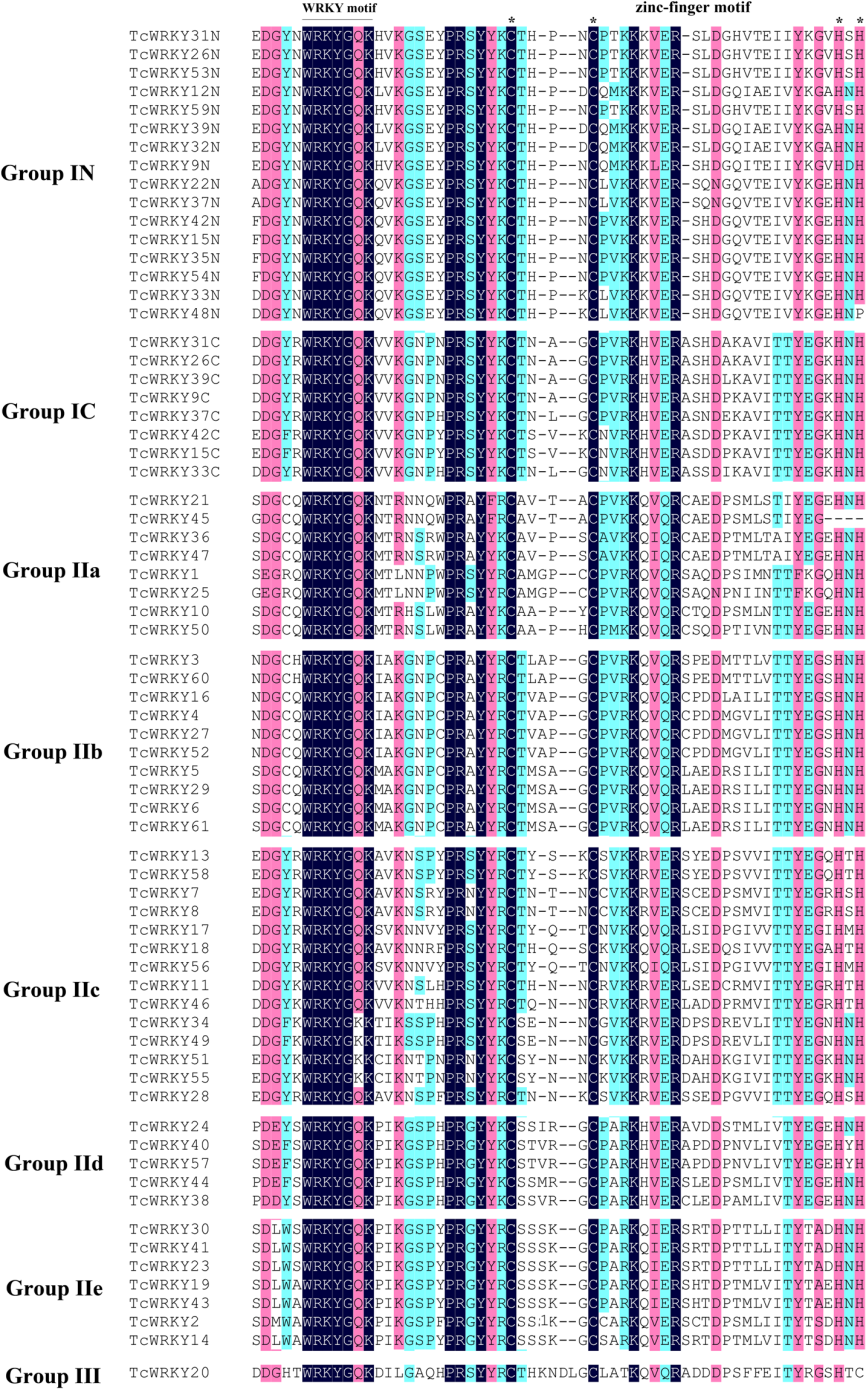
Sequence alignment of WRKY domain of 61 TcWRKY proteins. WRKYGQK motif was underlined as WRKY domain, and C_2_H(H/C) of zinc-finger motif was designated by star (*). TcWRKY45 was uncomplete so that its HxH residues were not found. All TcWRKYs were groups according to phylogenetic analysis. The names with N or C represented for N- and C-terminal WRKY domain of Group I respectively. Several Group I TcWRKYs didn’t find their C-terminal WRKY domain. Sequence alignment was conducted by ClustalW, the figure was generated by DNAMAN 6.0. Different colors indicate sequence similarities: black indicated 100%, purple is 75%, green is 50%.

The WRKY domain, which determines the molecular structures and functions of all WRKY proteins, consisted of the WRKYGQK and zinc finger motifs. The WRKYGQK motif was conserved in almost all TcWRKY factors, except for several subgroup IIc WRKYs, which was WRKYGKK instead in TcWRKY34, TcWRKY49, TcWRKY51, and TcWRKY55 (Fig. 2). The WRKYGKK sequence is the most common variant present in Group IIc not only in *Taxus* but also in soybean, *Solanum lycopersicum*^28^, *Lotus japonicus*^29^, and *Brassica oleracea* var. *capitata*^30,31^.

### Conserved domains of TcWRKY proteins

In addition to the conserved domains/motifs, WRKY proteins contain many diverse conserved domains, such as B3 domain, NAC (named after NAM, ATAF1/2, and CUC2 proteins) domain, zinc finger_SQUAMOSA promoter binding protein, Toll–interleukin receptor (TIR), leucine-rich repeat (LRR), paired amphipathic helix repeat (PAH), cystathionine-β-synthase (CBS), and kinase domain^11^.

**Figure 3:**
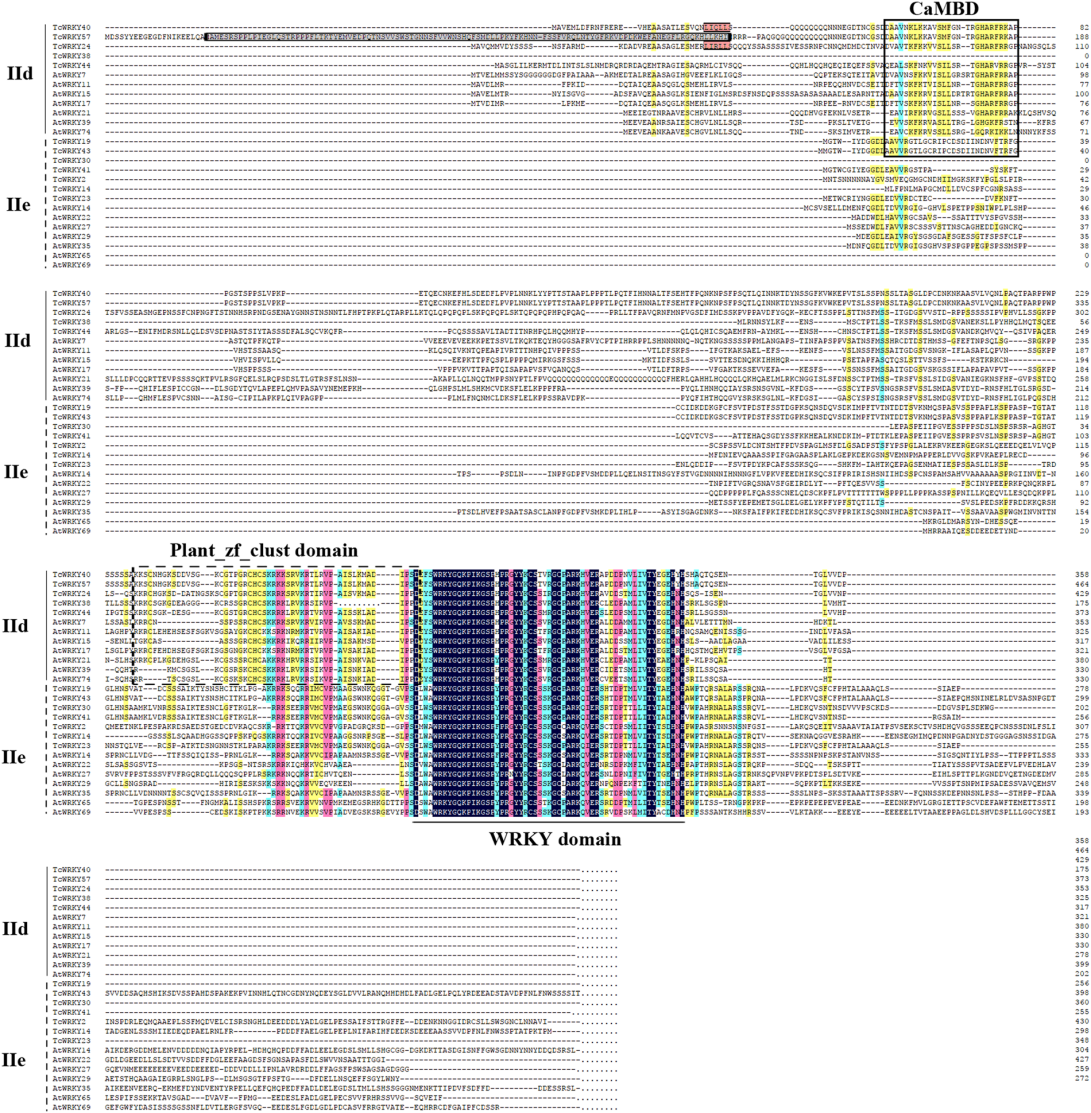
Alignment of Group IId and Group IIe WRKYs in *T*. *chinensis* and *A*. *thaliana*. Full-length WRKYs of Group IId and Group IIe were aligned together since they were considered as IId + e subgroup. WRKY domain was labeled by the solid line. CaMBD (Ca^2 +^-dependent CaM-binding domain) and Plant_zf_clust (plant zinc finger cluster, accession number in pfam was PF10533) domain were framed by solid box and dotted box respectively. HSF_DNA-bind (HSF-type DNA-binding, pfam00447) domain of TcWRKY57 was framed by grey box. LxxLL-motif was framed by red box. Sequence alignment was conducted by ClustalW, the figure was generated by DNAMAN 6.0. Different colors indicate sequence similarities: black indicated 100%, purple is 75%, green is 50%, yellow is 33%.

Aside from the Ca^2+^-dependent CaM-binding domain (CaMBD), all of Group IId TcWRKYs and AtWRKYs also contain the plant zinc finger cluster (Plant_zf_clust, PF10533) domain (Fig. 3). This result is in accordance with previous reports^32,33^. Group IIe and IId are commonly considered one subgroup, although Group IIe has no CaMBD. According to our results, Group IIe contains a peptide that is highly similar to the Plant_zf_clust domain of Group IId, such that they share similar functions and relationships (Fig. 3). TcWRKY57 also contains an HSF-type DNA-binding (HSF_DNA-bind, pfam00447) domain at its N-terminal (44 aa-133 aa) (Fig. 3).

**Figure 4:**
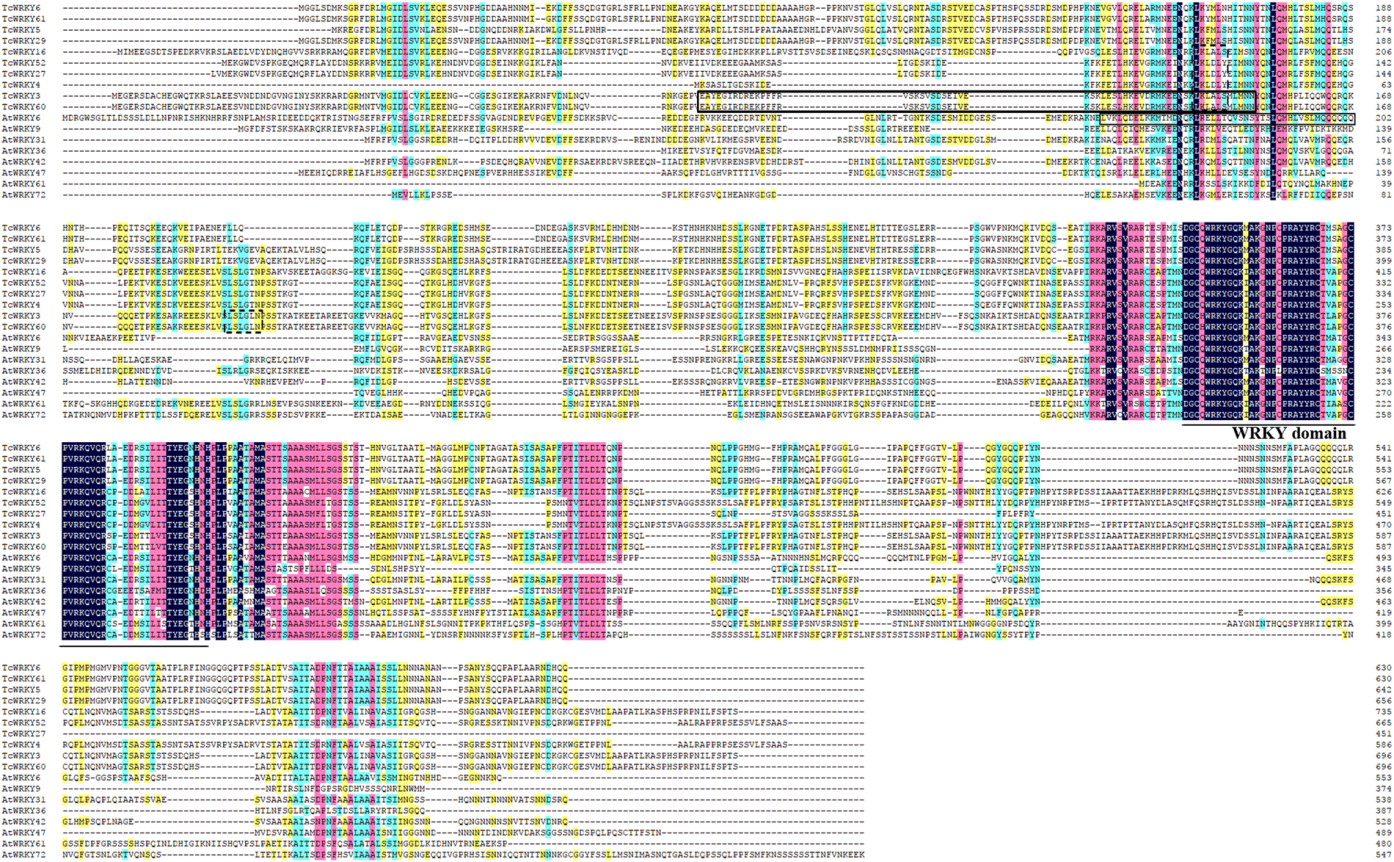
Alignment of Group IIb TcWRKYs and AtWRKYs. WRKY domain was labeled by the solid line. Leucine zipper motif (Accession NO.: pfam15294 in pfam) of TcWRKY3/60 were annotated by NCBI blastp (https://blast.ncbi.nlm.nih.gov/Blast.cgi) and labeled by solid box. Leucine zipper motif of AtWRKY6 also in solid box was the result of Robatzek *et al*.^36^. The EAR-motif was labeled by dotted boxes. Sequence alignment was conducted by ClustalW, the figure was generated by DNAMAN 6.0. Different colors indicate sequence similarities: black indicated 100%, purple is 75%, green is 50%, yellow is 33%.

TcWRKY24 (Group IId) contains the HARF (RTGHARFRR [A/G] P) motif, whose function has not been clearly determined, in its CaMBD (Fig. 3)^34^. Several TcWRKY proteins also contain the LxxLL motif, which participates in many protein–protein interactions associated with different aspects of transcriptional regulation, i.e., TcWRKY12 (LSQLL, Group I), TcWRKY20 (LYQLL, Group III), TcWRKY24 (LIRLL, Group IId), TcWRKY32 (LVRLL, Group I), TcWRKY33 (LSPLL, Group I), TcWRKY40 (LIQLL, Group IId), and TcWRKY48 (LSPLL, Group I) (Fig. 3, supplement S2)^35^. The NCBI BLASTP results showed that TcWRKY3 and TcWRKY60 contain a leucine zipper (pfam15294) domain at the N-terminal (93 aa-147 aa), which is also detected in AtWRKY6 (IIb) (Fig. 4b)^36^.

Overall, some novel conserved domains were detected in TcWRKY proteins. However, current conserved domains detected in WRKY proteins, such as LRR, were not identified in TcWRKYs. Moreover, completed sequences need to be further identified to clarify the molecular structure of WRKYs in *Taxus*.

### Group IIb TcWRKY proteins contain EAR motifs

Notably, several Group IIb TcWRKYs contain the ERF-associated amphiphilic repression (EAR) motif, which is a strong repression domain present in various repressors^37^. TcWRKY4/27/52 has the LKLDLY-type EAR motif, whereas TcWRKY3/16/60 has the LKLALS-type EAR motif. TcWRKY3/60 also has an LSLGLN-type EAR motif at the C-terminal of the LKLDLY-type EAR motif (Fig. 4).

Several AtWRKYs also contain the EAR motif, i.e., AtWRKY14 (IIe) and AtWRKY18 (IIa) had the DLNxNP-type EAR motif, whereas AtWRKY9 (IIb) has two LSLSL-type EAR motifs^38^. According to our results, AtWRKY36 (IIb) contains the LKLLLS-type EAR motif (Fig. 4). Notably, no Group IIa or IIe TcWRKYs contains any EAR motif.

### Expression profiles of *TcWRKY* genes

**Figure 5:**
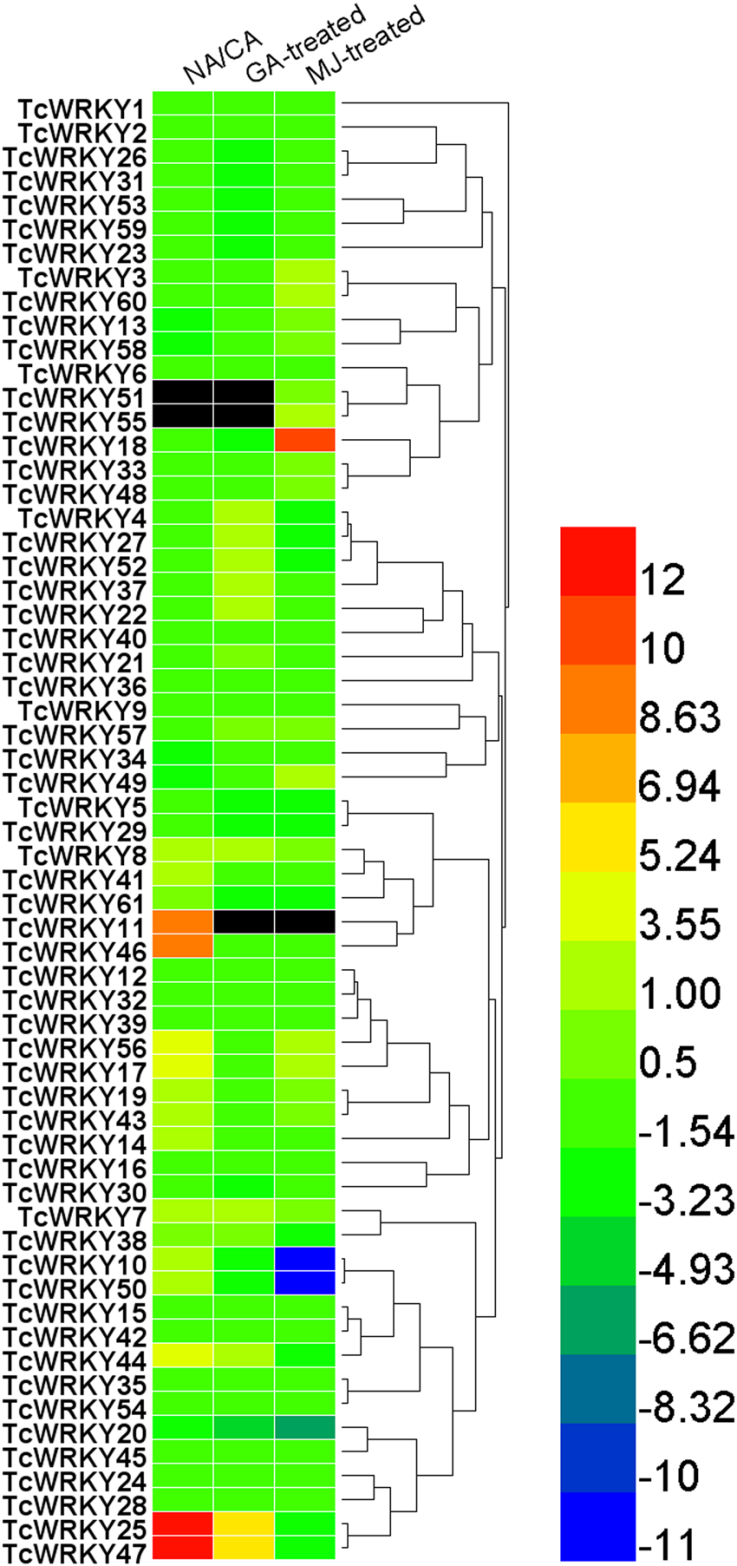
Expression profiles of 61 *TcWRKYs*. Expression profiles of 61 TcWRKYs were analyzed according to three transcriptome datasets. NA was newly isolated *Taxus* cells while CA was 10-years long-term subcultured cells, the taxol content between the two samples was highly different (Zhang *et al*., 2015)^21^. MeJA (methyl jasmonate acid)-treated (Li *et al*., 2012)^20^ and GA (gibberellin)-treated (unpublished work) mean the cells were treated by MeJA for 15 h and GA for 6 h respectively. The heatmap was generated by Heml 1.0 software (http://hemi.biocuckoo.org/). The black boxes indicated that these *TcWRKYs* were not found in the dataset.

A hierarchical cluster analysis was performed using the three experimental datasets to determine the potential roles of the 61 *TcWRKY* genes in plant responses to various environmental stresses (Fig. 5). NA denotes the newly isolated cells, whereas CA denotes the 10 year long-term subcultured cells; the taxol content between these two samples was highly different^21^. Methyl jasmonate acid (MeJA) and gibberellin (GA) are plant endogenous hormones^20^.

Most TcWRKYs changed significantly in the three experimental datasets (Fig. 5). Of 61 TcWRKYs, 50 were changed significantly, 43 were downregulated after MeJA treatment for 15 h, and only TcWRKY3/60 (Group IIb) and TcWRKY17/18/49/55/56 (Group IIc) were increased. Of 55 differentially expressed (DE) TcWRKYs, 45 were downregulated after GA treatment for 3 h. TcWRKY4/27/52 (Group IIb), TcWRKY7/8 (Group IIc), TcWRKY22/37 (Group I), TcWRKY44 (Group IId), and TcWRKY25/36 (Group IIa) were induced by GA treatment. Of 57 DE TcWRKYs, 42 were downregulated in NA than in CA. TcWRKY7/8/11/17/46/56 (Group IIc), TcWRKY10/25/50/36 (Group IIa), TcWRKY14/19/43/41 (Group IIe), and TcWRKY44 (Group IId) were upregulated in NA.

### Expression patterns of selected TcWRKYs induced by SA and MeJA

A total of seven TcWRKYs, one from each subgroup, i.e., TcWRKY26 (Group I), TcWRKY47 (Group IIa), TcWRKY52 (Group IIb), TcWRKY8 (Group IIc), TcWRKY44 (Group IId), TcWRKY41 (Group IIe), and TcWRKY20 (Group III), were selected. The expression patterns of these TcWRKY genes responding to MeJA and SA treatments were determined. Results showed that the seven TcWRKYs had different response patterns to MeJA and SA treatments.

**Figure 6:**
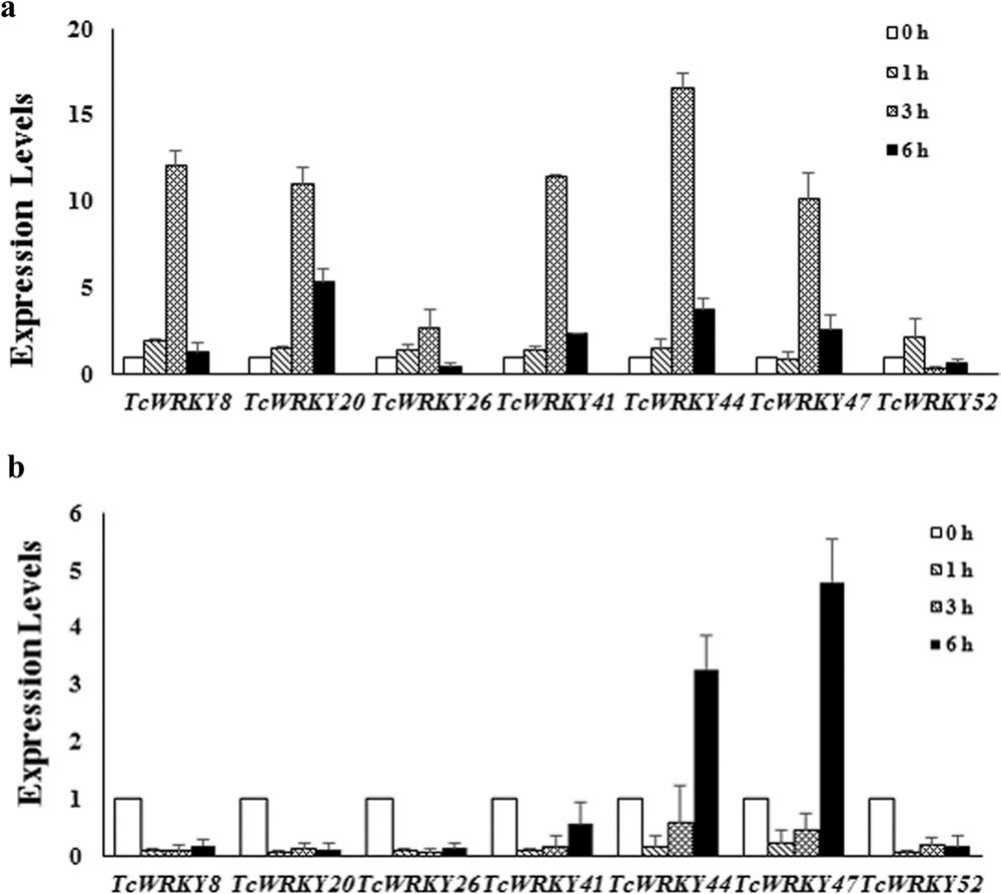
Expression patterns of selected *TcWRKYs* in response to MeJA and SA. Seven TcWRKYs, one from each subgroup, were selected to clarify the expression patterns in response to (**a**) MeJA (Methyl Jasmonate acid) and (**b**) SA (Salicylic acid). Expression levels of *TcWRKY8* (Group IIc), *TcWRKY20* (III), *TcWRKY26* (I), *TcWRKY41* (IIe), *TcWRKY44* (IId), *TcWRKY47* (IIa) and *TcWRKY52* (IIb) were determined by qRT-PCR. Actin was used as reference gene, and each experiment was conducted three repeats.

After MeJA treatment, *TcWRKY8*, *TcWRKY20*, *TcWRKY26*, *TcWRKY41*, *TcWRKY44*, and *TcWRKY47* significantly increased at 3 h, reaching 12.1, 11.0, 2.6, 11.2, 16.5, and 10.1 times, respectively. Meanwhile, at 6 h, their expression levels decreased in varying degrees, with only *TcWRKY20* and *TcWRKY47* remaining >2 times higher than the control. Moreover, *TcWRKY52* was insensitive to MeJA treatment (Fig. 6a). *TcWRKY8*, *TcWRKY20*, *TcWRKY26*, *TcWRKY41*, and *TcWRKY52* were significantly inhibited at 6 h after SA treatment. By contrast, although *TcWRKY44* and *TcWRKY47* were reduced at 3 h, they were significantly enhanced at 6 h by 3.3 and 4.8 times, respectively (Fig. 6b). Overall, only *TcWRKY44* and *TcWRKY47* were induced by MeJA and SA treatments.

### Overexpression of TcWRKYs

**Figure 7:**
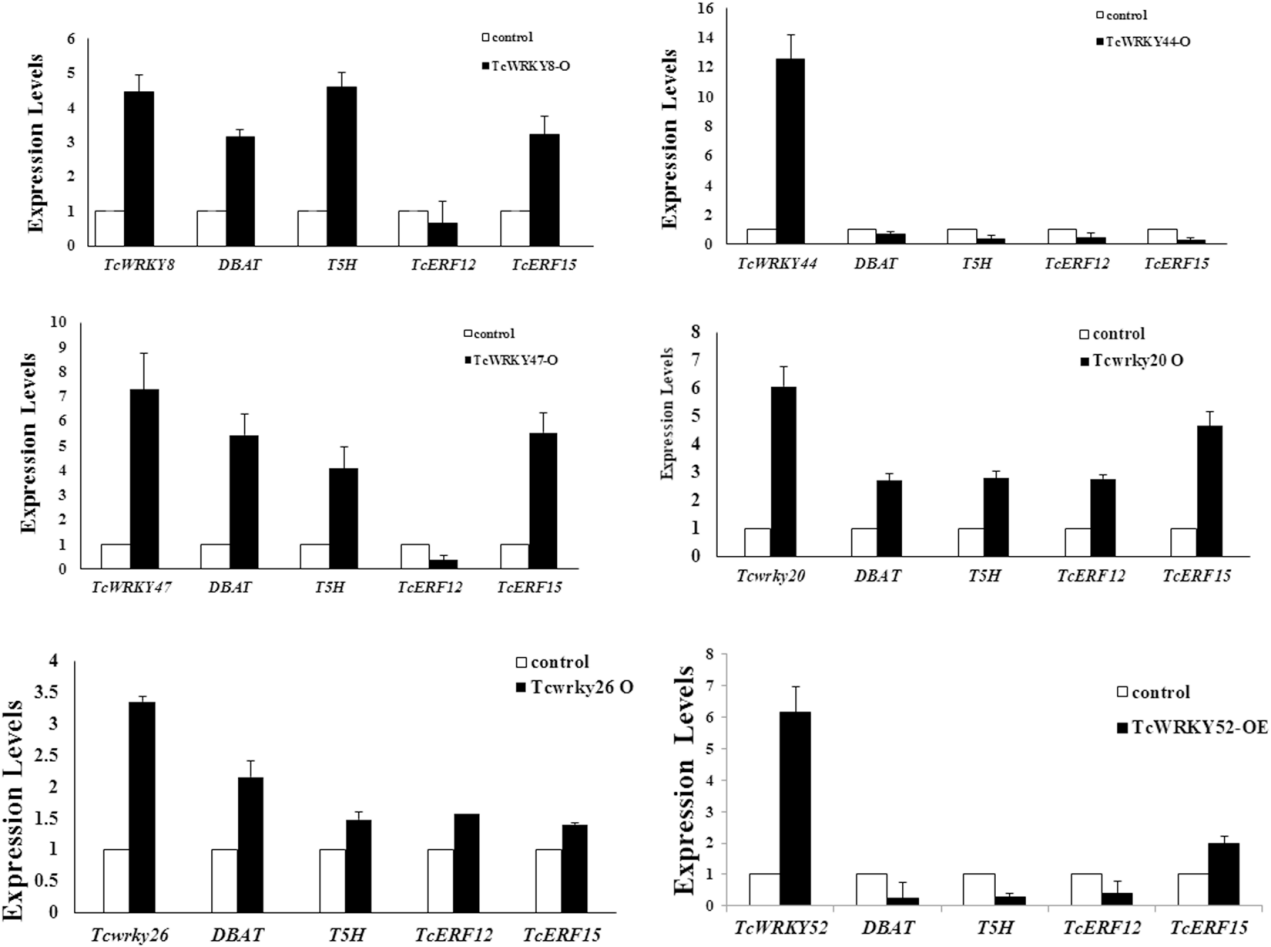
Expression profiles of taxol-biosynthesis-related genes in six overexpression cell lines. Expression of *DBAT* and *T5H* genes, which encoded taxol biosynthesis enzymes, were certificated to be controlled by W-boxes in their promoters. Promoter of *TcERF12* and *TcERF15*, which were regulators of taxol biosynthesis, also contained W-boxes. The expression levels of taxol-biosynthesis-related genes were determined by qRT-PCR. *Actin* was used as reference gene, and each experiment was conducted three repeats.

According to previous reports, Group IId and IIe were grouped together as Group IId + e and verified to function redundantly in other plants^13^. Therefore, TcWRKY44 was selected for further studies to test the functions of Group IId + e WRKYs on taxol biosynthesis. Then, six TcWRKYs were overexpressed in the *T*. *chinensis* cell lines. Our previous research verified W-box as the key *cis*-element of promoters of *DBAT*^6^ and *T5H*. *TcERF12* and *TcERF15*, which encode regulators of taxol biosynthesis, also contain W-boxes in their promoters (unpublished work). When these TcWRKYs are overexpressed, the expression levels of *DBAT*, *T5H*, *TcERF12*, and *TcERF15* change in varying degrees (Fig. 7).

*TcWRKY8*, *TcWRKY20*, *TcWRKY47*, and *TcWRKY26* could increase the expression levels of the *DBAT* (3.1, 2.7, 5.4, and 2.2 times), *T5H* (4.6, 2.8, 4.1, and 1.5 times), and *TcERF15* (3.2, 4.7, 5.5, and 1.4 times) genes. However, only TcWRKY20 and TcWRKY26 could also increase *TcERF12* by 2.8 times. By contrast, TcWRKY47 and TcWRKY8 inhibit *TcERF12* by 0.4 and 0.8 times, respectively.

Meanwhile, TcWRKY44 and TcWRKY52 inhibit the expression levels of the *DBAT* (0.7 and 0.2 times), *T5H* (0.4 and 0.3 times), and *TcERF12* (0.5 and 0.4 times) genes significantly. TcERF15 is also downregulated by 0.3 times in TcWRKY44-overexpressing cell lines but upregulated by 2.0 times in TcWRKY52-overexpressing cell lines. The results show that the overexpression of different TcWRKYs exerts different effects on the expression of taxol-biosynthesis-related genes.

## Discussion

WRKY, the most pivotal transcription factors in plants, could regulate the expression of downstream genes by combining with *cis*-acting element W-box^39^. WRKY plays indispensable roles in regulating various physiological processes, including biotic and abiotic stress responses, signal molecule delivery, plant senescence, and synthesis of secondary metabolites. Taxol is a precious secondary metabolite initially isolated from *Taxus* spp. and widely used as an anticancer drug. Previous studies on promoters of taxol biosynthesis genes suggested that WRKY transcription factors play important regulatory roles in taxol biosynthesis^6,20,23^. Therefore, we conducted a systematic research on WRKY transcription factors of *T*. *chinensis*.

In the present study, 61 TcWRKYs were identified from the *T*. *chinensis* transcriptome datasets. Meanwhile, 75 TcWRKYs were identified in *Arabidopsis*, 109 in *O*. *sativa*^9^, 83 in *Pinus monticola*^40^, 62 in *Picea abies*^11^. Identifying all WRKYs from the transcriptome datasets is difficult because of the limited genome information of *Taxus*. For instance, *P*. *abies*, whose genome has been sequenced, is also a conifer and considered the most relative species of *Taxus* spp. Therefore, 61 TcWRKYs should comprise almost the entire WRKY factor family of *Taxus* compared with the 62 WRKYs of *P*. *abies*. All of these results indicated that we have identified almost all WRKYs of *T*. *chinensis*, such that the phylogenetic and functional analyses were highly representative.

On the basis of our results, all WRKY proteins from *T*. *chinensis* and *A*. *thaliana* were cladded into eight subgroups (i.e., Group I, IIa-IIe, IIg, and III) by their WRKY domains. Group IIf and IIg are not widespread WRKY proteins, such that Group IIf does not exist in *Arabidopsis* and cotton, whereas Group IIg does not exist in physic nut and rice; however, these WRKYs contain an additional MAP/ERK kinase kinase domain or TIR-NBS-LRR (NBS-LRR short for nucleotide-binding site leucine-rich repeat) domain^26,41^. Currently, some WRKY proteins were determined to contain three or four WRKY domains, whereas no such WRKYs were detected in *T*. *chinensis* in our work^11^.

In general, WRKY proteins could be classified by a particular WRKY domain, which mainly consists of 60 highly conversed amino acid sequences and has a WRKYGQK motif in the N-terminus^42^. Sometimes, the core sequence can be mutated into WRKYGKK^43^, which is the most common variant in soybean, *S*. *lycopersicum*^28^, *L*. *japonicus*^29^, and *B*. *oleracea* var. *capitata*^30^ and has the highest frequency in Group IIc. In the present study, TcWRKY34, TcWRKY49, TcWRKY51, and TcWRKY54, all of which belong to Group IIc, contain the WRKYGKK motif. In tobacco, the WRKYGKK sequence could bind specifically to the WK-box (TTTTCCAC), which is significantly different from the consensus sequence of W-box, indicating that the four TcWRKY proteins have similar functions in *Taxus*^44^. In addition to the WRKYGKK sequence, other heptapeptide variants in the WRKY domains exist in many plants. For example, WRKYGEK, WRKYGKR, WRKYEDK, WKKYGQK, and WHQYGLK variants were detected in the WRKY domains of *Glycine max* var. Williams 82^31^. According to our results and prior knowledge, no such variants have been identified in either *Taxus* or *P*. *abies*, indicating that these variants should be specific in angiosperm plants.

HARF, Leu-zipper, LxxLL, and EAR motifs were commonly detected in WRKY proteins, whereas TIR, LRR, PAH, and CBS domains were rarely identified. In *Taxus*, WRKY proteins contain HARF, Leu-zipper, LxxLL, and EAR motifs, indicating that these motifs of TcWRKYs serve similar functions to AtWRKYs. The HSF domain was also identified in TcWRKY proteins, and the influence of these domains needs to be further clarified. Moreover, verifying the details of the molecular structural information of TcWRKY proteins are difficult because of the limited genome information of *Taxus* spp.

WRKY proteins from different subgroups play either positive or negative roles; some of them even play dual roles in regulating downstream gene expression levels^13^. Thus, six TcWRKYs, i.e., TcWRKY8 (Group IIc), TcWRKY20 (Group III), TcWRKY26 (Group I), TcWRKY44 (Group IId + e), TcWRKY47 (Group IIa), and TcWRKY52 (Group IIb), were selected to verify their own functions on taxol biosynthesis. Then, these six TcWRKYs were overexpressed in *T*. *chinensis* cells, and *DBAT*, *T5H*, *TcERF15*, and *TcERF12*, all of which are taxol-biosynthesis-related genes, were analyzed by qRT-PCR.

TcWRKY44 (Group IId + e) and TcWRKY52 (Group IIb) suppressed the expression of the four genes, indicating that they are putative negative regulators of taxol biosynthesis. To our knowledge, AtWRKY7/11/17, which are all Group IId WRKYs, serve as negative defense regulators^45,46^. Meanwhile, AtWRKY6 (Group IIb) could play dual roles, i.e., a negative regulator in low Pi stress and a positive regulator in low B stress^47–49^. TcWRKY52 contains an EAR motif, resulting in its negative roles in regulating taxol biosynthesis.

By contrast, TcWRKY26 (Group I) and TcWRKY20 (Group III) improve the expression of four taxol-biosynthesis-related genes. Actually, Group III WRKYs always play positive roles in regulating the biosynthesis of secondary metabolites. For instance, CrWRKY1 and AaWRKY1, both of which belong to Group III, could improve the biosynthesis of vinblastine in *Catharanthus roseus* and artemisinin in *A*. *annua*, respectively^17,50^. Moreover, AtWRKY25, AtWRKY26, and AtWRKY33, which are Group I WRKYs in *Arabidopsis*, could upregulate the expression of *HsfA2*, *HsfB1*, *Hsp101*, and *MBF1c*^51^. In *Capsicum annuum*, CaWRKY58 acts as a transcriptional activator of negative regulators in the resistance of pepper to *Ralstonia solanacearum* infection^52^. All of these results indicate that TcWRKY26 and TcWRKY20 are positive regulators in *T*. *chinensis*.

Our results showed that TcWRKY8 (Group IIc) and TcWRKY47 (Group IIa) upregulate the expression of the *DBAT*, *T5H*, and *TcERF15* genes but downregulate the expression of *TcERF12*. TcERF12 is a negative regulator of the *TASY* gene, which encodes one of the key taxol biosynthesis enzymes. TcWRKY8 is a Group IIc WRKY protein with the typical WRKYGQK motif, whereas TcWRKY47 is a Group IIa WRKY protein. WRKY proteins from these two subtypes are reported as positive or negative regulators. For instance, GaWRKY1 and OsWRKY62 are Group IIa WRKYs, but GaWRKY1 positively regulates the sesquiterpene synthase gene (+)-δ-cadinene synthase-A in cotton, whereas OsWRKY62 negatively regulates the basal and Xa21-mediated defense against *Xanthomonas oryzae*^16,53^.

In summary, the 61 WRKY proteins from the *T*. *chinensis* transcriptome datasets were highly representative and adequate for further research. Phylogenetic analysis of the WRKY domains showed that the TcWRKYs of *T*. *chinensis* could be divided into Group I, IIa–IIe, and III as well as those of *Arabidopsis*, indicating that the WRKY transcription factors exhibit species divergence. Further overexpression of TcWRKY8/20/26/44/47/52 indicated the diverse functions of TcWRKY factors in *Taxus* and identified candidate regulators of taxol biosynthesis.

### Data availability

All the protein sequences of TcWRKYs were included in Supplementary file 2. The AtWRKYs were obtained from TAIR (http://www.arabidopsis.org/browse/genefamily/WRKY-Som.jsp).

## Electronic supplementary material

Supplementary table and dataset legends

Supplementary material file 2
