# Supplementary material for: Transcriptome-wide identification and screening of WRKY factors involved in the regulation of taxol biosynthesis in Taxus chinensis
Source: Sci Rep. 2018 Mar 26;8:5197. doi: 10.1038/s41598-018-23558-1 (PMC5980082; doi:10.1038/s41598-018-23558-1)
Supplement: Supplementary file 1 — Supplementary table and dataset legends [file 41598_2018_23558_MOESM1_ESM.docx]

**Supplementary Information**

**Transcriptome-wide identification and screening of WRKY factors involved in the regulation of taxol biosynthesis in *Taxus chinensis***

Meng Zhang ^1, 2^, Ying Chen ^1, 2, #^, Lin Nie^1, 2^, Xiaofei Jin^1, 2^, Weifang Liao^1, 2^, Shengying Zhao^1, 2^, Chunhua Fu^1, 2, *^, Longjiang Yu^1, 2^

1 Institute of Resource Biology and Biotechnology, Department of Biotechnology, College of Life Science and Technology, Huazhong University of Science and Technology, No.1037 Luoyu Road, Wuhan, 430074, P. R. China

2 Key Laboratory of Molecular Biophysics Ministry of Education, College of Life Science and Technology, Huazhong University of Science and Technology, No.1037 Luoyu Road, Wuhan, 430074, P. R. China

# indicates the author’s work equals to first author

* Corresponding author

Tel/Fax: +86 (0)27- 87792432, E-mail: fuch2003@126.com

| **Supplementary Table S1 Primers used in this paper** | | |
| --- | --- | --- |
|  |  |  |
| Name | Primer sequence | Restriction site |
| *TcWRKY8*F | CCCGGGATGGCAGGCCAGCAGCCT | *Sma* I |
| *TcWRKY8*R | GAGCTCTTATTTGCGCATTCCCGGT | *Sac* I |
| *TcWRKY20*F | ACTAGTATGGAGATAGGGGAGGGGAAA | *Spe* I |
| *TcWRKY20*R | GGTNACCCTAAAAAGGATCGCTTCTCTCAA | *Bst*E II |
| *TcWRKY26*F | GGATCCATGGAGGGCGGAAACAATGTTA | *Bam*H I |
| *TcWRKY26*R | GAGCTCTCAAGGGCCCATAAGTAATCT | *Sac* I |
| *TcWRKY41*F | GGATCCATGGGAACCTGGTGCG | *Bam*H I |
| *TcWRKY41*R | GAGCTCTCATGAATTGGGCATTT | *Sac* I |
| *TcWRKY44*F | ACTAGTATGTTAAGGAACAATTCCTA | *Spe* I |
| *TcWRKY44*R | GGTNACCTTAAGTATGCATTACAAGAT | *BstE* II |
| *TcWRKY47*F | CCCGGGATGGACCAATGCAAAAAT | *Sma* I |
| *TcWRKY47*R | GAGCTCTTAGCTACACATACCTT | *Sac* I |
| *TcWRKY52*F | GGATCCATGAAGTCTGCATCTCTCACC | *Bam*H I |
| *TcWRKY52*R | GAGCTCTCAAGAAGCAGCTGAAAATAGGA | *Sac* I |
| dlActinF | TCCTGCTTTGCTCACT |  |
| dlActinR | CCTCATCACCGACATA |  |
| dl*TcWRKY8*F | TCTGCTGGAAGACGGGTAT |  |
| dl*TcWRKY8*R | TGGCATGGATGGGTGC |  |
| dl*TcWRKY20*F | GGGCTCAGGGTTGTTT |  |
| dl*TcWRKY20*R | CTCGGCGTTCATTGTT |  |
| dl*TcWRKY26*F | GGAGACGGCTCAACCA |  |
| dl*TcWRKY26*R | GCCTGCCAATACCTCG |  |
| dl*TcWRKY41*F | GCAGAAGGGTCGTGGAA |  |
| dl*TcWRKY41*R | TCGTGCGAGTGCGTTT |  |
| dl*TcWRKY44*F | CAGGGTCTTCCAAACAA |  |
| dl*TcWRKY44*R | AGAGTCCCAGCCATCA |  |
| dl*TcWRKY47*F | TGAAGACCCAACTATGC |  |
| dl*TcWRKY47*R | ATGCTGGAAACGGAAG |  |
| dl*TcWRKY52*F | AACTGCCACCATTACA |  |
| dl*TcWRKY52*R | GATTCAGAGGGACGAG |  |
| dl*DBAT*F | CATGGCTGACACTGACCTCT |  |
| dl*DBAT*R | CCTGCTCCTAGTCCATCACA |  |
| dl*T5H*F | GCGCTGCAGAGTTACATTG |  |
| dl*T5H*R | AGACGATCCTGTTCCTGCTT |  |
| dl*TcERF12*F | GCGACGGATGGATTTA |  |
| dl*TcERF12*R | GTTTGGCTCGGCACTT |  |
| dl*TcERF15*F | CCATCTCCTGCTCGTC |  |
| dl*TcERF15*R | AACACTGGGTTGGTCTG |  |

Note: The primers names started with dl were used for qRT-PCR. The other primers were used for overexpression vectors, and the restriction sites indicated the restriction enzymes used in vector constructing. BamH I, Sma Iand Sac I were used in pBI121; Spe I and BstE II were used in pCAMBIA1303.

**Supplementary datasets legends**

**Supplementary material file 2 Protein sequences of 61 TcWRKYs**

61 protein sequences included full-length and incomplete TcWRKYs. Full-length TcWRKYs were obtained by ORF Finder (https://www.ncbi.nlm.nih.gov/orffinder/), and incomplete sequences were the aligned part after online blastx programme against NR (non-redundant) database (https://blast.ncbi.nlm.nih.gov/Blast.cgi).
